# Dissecting Listeria monocytogenes Persistent Contamination in a Retail Market Using Whole-Genome Sequencing

**DOI:** 10.1128/spectrum.00185-22

**Published:** 2022-05-17

**Authors:** Yan Wang, Lijuan Luo, Shunshi Ji, Qun Li, Hong Wang, Zhendong Zhang, Pan Mao, Hui Sun, Lingling Li, Yiqian Wang, Jianguo Xu, Ruiting Lan, Changyun Ye

**Affiliations:** a State Key Laboratory of Infectious Disease Prevention and Control, National Institute for Communicable Disease Control and Prevention, Collaborative Innovation Center for Diagnosis and Treatment of Infectious Diseases, Chinese Center for Disease Control and Prevention, Beijing, China; b School of Biotechnology and Biomolecular Sciences, University of New South Wales, Sydney, Australia; c Zigong Center for Disease Control and Prevention, Zigong, Sichuan Province, China; University of California Davis

**Keywords:** *Listeria monocytogenes*, CC87, CC87-wg-MLST, persistent contamination, whole-genome sequencing, retail market, meat stall, aquatic foods stall

## Abstract

Listeria monocytogenes is a foodborne pathogen that can cause invasive disease with high mortality in immunocompromised individuals and can survive in a variety of food-associated environments for a long time. L. monocytogenes clonal complex (CC) 87 is composed of ST87 and three other STs and has been identified as the most common subgroup associated with both foods and human clinical infections in China. Therefore, the persistence of CC87 L. monocytogenes in food-associated environments poses a significant concern for food safety. In this study, 83 draft genomes of CC87 L. monocytogenes, including 60 newly sequenced genomes, were analyzed with all isolates from our previous surveillance in Zigong, Sichuang, China. Sixty-eight of the studied isolates were isolated from one retail market (M1 market), while the others were from seven other markets (M2–M8 markets) in the same city. Whole-genome multilocus sequence typing (wg-MLST) and the whole-genome single nucleotide polymorphism (wg-SNP) analysis were performed. Three persistent contamination routes were identified in the M1 market, caused by 2 clusters (A and B) and a wgST31 type. Cluster A isolates were associated with the persistent contamination in a raw meat stall (M1-S77), while Cluster B isolates caused a persistent contamination in aquatic foods stalls. Five wgST31 isolates caused persistent contamination in a single aquatic stall (M1-S65). A pLM1686-like plasmid was found in all Cluster A isolates. A novel plasmid, pLM1692, a truncated pLM1686 plasmid without the cadmium, and other heavy metal resistance genes were conserved in all wgST31 isolates. By comparing persistent and putative non-persistent isolates, four genes that were all located in the prophage comK might be associated with persistence. These findings enhanced our understanding of the underlying mechanisms of contamination and assist in formulating targeted strategies for the prevention and control of L. monocytogenes transmission from the food processing chain to humans.

**IMPORTANCE** Contamination of food by Listeria monocytogenes at retail level leads to potential consumption of contaminated food with high risk of human infection. Our previous study found persistent contamination of CC87 L. monocytogenes from a retail market in China through pulsed-field gel electrophoresis and multilocus sequence typing. In this study, whole-genome sequencing was used to obtain the highest resolution inference of the source and reasons for persistent contamination; meat grinders and minced meat were the major reservoir of persistent contamination in meat stalls, whereas fishponds were the major reservoir in seafood stalls, with different L. monocytogenes isolates involved. These isolates carried different properties such as plasmids and prophages, which may have contributed to their ability to survive or adapt to the different environments. Our findings suggest that whole-genome sequencing will be an effective surveillance tool to detect persistent L. monocytogenes contamination in retail food markets and to design new control strategies to improve food safety.

## INTRODUCTION

Listeria monocytogenes is an opportunistic pathogen responsible for foodborne illness with a variety of manifestations from mild gastroenteritis to severe invasive infections (1). Invasive listeriosis is dangerous for immunocompromised individuals leading to septicemia, meningitis, and encephalitis (2). Consumption of contaminated food, such as meat, fish, dairy, and ready-to-eat food is the major route of human infection (3, 4). L. monocytogenes is widespread in a range of environments due to its ability to survive and grow under harsh environmental conditions, such as low pH, low temperatures, and high osmolarity (5). L. monocytogenes introduced into a food-associated environment increases the risks of establishing contaminated niches followed by adherence and colonization leading to persistent contamination.

The population of L. monocytogenes has been found comprised of four genetic groups with Lineages I to IV, which are further divided by different sequence types (STs) by multilocus sequence typing (MLST), in which closely related STs are classified as a clonal complex (CC) (6). ST87 had been identified as a prevalent sequence type of L. monocytogenes of different origins in China (7–9). Together with 3 other STs (namely ST305, ST310, and ST1166), ST87 forms the CC87 clonal complex. CC87 isolates have been rarely reported in Europe and the U.S., except for an outbreak in Spain (10). Recently, subtyping schemes using whole-genome sequences, such as whole-genome multilocus sequence typing (wg-MLST) and whole-genome single nucleotide polymorphism (wg-SNP), have been recognized as effective tools for providing better understandings of the patterns of persistence and dissemination for L. monocytogenes within a certain niche or during an outbreak (11–13).

ST87 L. monocytogenes is commonly isolated from food products, natural environments, and sporadic listeriosis in China (8, 14, 15). Moreover, this subgroup of L. monocytogenes has been gradually recognized as most commonly causing listeriosis in China, with the prevalence rate up to 34% (8, 16, 17). In our previous study we examined the genomic features and the population structure of ST87 L. monocytogenes from different regions of China (18). The genomes of ST87 L. monocytogenes represent a highly conserved and stable backbone with few accessory genes, which are mainly located in mobile genetic elements, such as prophages and plasmid. A conserved pLM1686-like plasmid with the size of 91 kb was found in a set of ST87 isolates (18). The plasmid carried heavy metal resistance genes, including *cadA1*, *cadA2*, *cadC*, and *copB*, which are related to adaptation to harsh environments (19, 20). We also conducted a retail market surveillance of L. monocytogenes for 12 months in 2015, with sampling once a month in Zigong, China (21). Food and environmental samples were collected from eight retail markets. The molecular characteristics and potential persistent contamination of L. monocytogenes from meat stalls were identified based on pulsed-field gel electrophoresis (PFGE) and MLST (21). Here, we performed an in-depth study on the CC87 L. monocytogenes isolates from the retail market surveillance by using whole-genome sequencing. A panel of 68 CC87 L. monocytogenes isolates in a retail market (M1) along with 15 isolates from seven other markets (M2-M8) were selected to (i) confirm the persistent contamination of CC87 L. monocytogenes in the retail market, (ii) probe the genetic variations among the persistent CC87 isolates over a year in different niches, (iii) better understand the contamination routes in the retail market, and (iv) explore potential genetic factors of L. monocytogenes persistence.

## RESULTS

### Selection of CC87 Listeria monocytogenes isolates for whole genome sequencing.

In our previous study of surveillance of retail markets, we identified persistent contamination by the same isolates through PFGE analysis. Here, based on PFGE profiles, we selected part of CC87 L. monocytogenes isolates for genomic analysis to further confirm and investigate the persistent contamination that occurred in a retail market (M1 market). Twenty Pulsotype-30 (PT-30) isolates associated with pork products and environments had already been published in Luo et al.’s study (21). Additionally, 43 isolates associated with aquatic products, other meat products (except for pork and aquatic products), and environments with PT-30 from the same market were used in this study. The surveillance of the aquatic products, other meat products, and environments was done at the same time as the study of Luo et al., and isolates obtained were typed similarly. Except for the isolates from pork stalls, there were 78 isolates obtained in the M1 market over the 12-month survey. By serotype, 57 were 1/2b, by ST, 13 ST87 and 40 ST1166 (both STs belonging to CC87). By PFGE, 50 isolates were PT30, 43 of which were selected for this study. The distribution of the isolates among the aquatic stalls and other meat stalls over the sampling months are presented in Fig. S1 in the supplemental material. For comparison, we chose two groups of isolates from the same surveillance: (i) 9 PT30 L. monocytogenes isolates from other markets (M2–M8), and (ii) 11 non-PT30 (PT27, PT315, PT317, PT331, PT322, and PT324) but CC87 isolates from all markets. The background information of each L. monocytogenes isolate is listed in Table 1. All the non-M1 markets were randomly named M2 to M8. Sixty isolates were subjected to sequencing in this study, and the other 23 isolates had been sequenced in our previous study (18). The complete genome of isolate ICDC-LM188 (accession no. CP015593), belonging to CC87/ST87, was used as a reference.

**TABLE 1:** Listeria monocytogenes isolates included in this study

Isolate name	Market and stall	Sampling mo and yr	PFGE profile	Sequence type	wg-MLST	Cluster
ICDC-LM1253	M1-S77	Feb, 2015	30	87	1	Cluster A
ICDC-LM1341	M1-S77	Mar, 2015	30	87	1	Cluster A
ICDC-LM1457	M1-S77	Apr, 2015	30	87	1	Cluster A
ICDC-LM1496	M1-S23	May, 2015	30	87	1	Cluster A
ICDC-LM1605	M1-S77	Jun, 2015	30	87	1	Cluster A
ICDC-LM1201	M2-S19	Nov, 2014	30	87	2	Cluster A
ICDC-LM1346	M1-S77	Mar, 2015	30	87	2	Cluster A
ICDC-LM1459	M1-S77	Apr, 2015	30	87	2	Cluster A
ICDC-LM1515	M1-S13	May, 2015	30	87	2	Cluster A
ICDC-LM1542	M1-S22	May, 2015	30	87	2	Cluster A
ICDC-LM1249	M1-S77	Feb, 2015	30	87	3	Cluster A
ICDC-LM1509	M1-S77	May, 2015	30	87	3	Cluster A
ICDC-LM1508	M1-S77	May, 2015	30	87	4	Cluster A
ICDC-LM1604	M1-S77	Jun, 2015	30	87	5	Cluster A
ICDC-LM1250	M1-S77	Feb, 2015	30	87	6	Cluster A
ICDC-LM1523	M1-S58	May, 2015	30	87	7	Cluster A
ICDC-LM1452	M1-S58	Apr, 2015	30	87	8	Cluster A
ICDC-LM1449	M1-S14	Apr, 2015	30	87	9	Cluster A
ICDC-LM1404	M1-S58	Mar, 2015	30	87	10	Cluster A
ICDC-LM1637	M1-S77	Jul, 2015	319	87	11	Cluster A
ICDC-LM1514	M1-S14	May, 2015	30	87	12	Cluster A
ICDC-LM1728	M1-S45	Oct, 2015	30	87	13	Cluster A
ICDC-LM1208	M1-S64	Jan, 2015	30	1166	14	Cluster B
ICDC-LM1234	M1-S60	Jan, 2015	30	1166	14	Cluster B
ICDC-LM1255	M1-S63	Feb, 2015	30	1166	14	Cluster B
ICDC-LM1378	M1-S61	Mar, 2015	30	1166	14	Cluster B
ICDC-LM1453	M1-S63	Apr, 2015	30	1166	14	Cluster B
ICDC-LM1473	M1-S61	Apr, 2015	30	1166	14	Cluster B
ICDC-LM1525	M1-S61	May, 2015	30	1166	14	Cluster B
ICDC-LM1598	M1-S61	Jun, 2015	30	1166	14	Cluster B
ICDC-LM1599	M1-S60	Jun, 2015	30	1166	14	Cluster B
ICDC-LM1602	M1-S63	Jun, 2015	30	1166	14	Cluster B
ICDC-LM1603	M1-S62	Jun, 2015	30	1166	14	Cluster B
ICDC-LM1621	M1-S62	Jul, 2015	30	1166	14	Cluster B
ICDC-LM1626	M1-S65	Jul, 2015	30	1166	14	Cluster B
ICDC-LM1638	M1-S63	Jul, 2015	30	1166	14	Cluster B
ICDC-LM1744	M1-S62	Oct, 2015	30	1166	14	Cluster B
ICDC-LM1798	M1-S63	Oct, 2015	30	1166	14	Cluster B
ICDC-LM1807	M1-S51	Nov, 2015	30	1166	14	Cluster B
ICDC-LM1821	M1-S60	Dec, 2015	30	1166	14	Cluster B
ICDC-LM1844	M1-S61	Jan, 2015	30	1166	14	Cluster B
ICDC-LM1207	M1-S63	Jan, 2015	30	1166	15	Cluster B
ICDC-LM1845	M1-S60	Jan, 2015	30	1166	16	Cluster B
ICDC-LM1405	M1-S62	Mar, 2015	30	1166	17	Cluster B
ICDC-LM1500	M1-S62	May, 2015	30	1166	18	Cluster B
ICDC-LM1507	M1-S63	May, 2015	30	1166	19	Cluster B
ICDC-LM1524	M1-S60	May, 2015	30	1166	20	Cluster B
ICDC-LM1627	M1-S60	Jul, 2015	30	1166	21	Cluster B
ICDC-LM1658	M1-S62	Aug, 2015	30	1166	22	Cluster B
ICDC-LM1661	M1-S63	Aug, 2015	30	1166	23	Cluster B
ICDC-LM1662	M1-S60	Aug, 2015	30	1166	24	Cluster B
ICDC-LM1746	M1-S60	Oct, 2015	30	1166	25	Cluster B
ICDC-LM1812	M1-S61	Nov, 2015	30	1166	26	Cluster B
ICDC-LM1822	M1-S61	Dec, 2015	30	1166	27	Cluster B
ICDC-LM1540	M1-S61	May, 2015	30	1166	28	Cluster B
ICDC-LM1342	M1-S63	Mar, 2015	30	1166	29	Cluster B
ICDC-LM1420	M1-S62	Apr, 2015	30	1166	30	Cluster B
ICDC-LM1692	M1-S65	Sep, 2015	30	87	31	Cluster C
ICDC-LM1693	M1-S65	Sep, 2015	30	87	31	Cluster C
ICDC-LM1713	M1-S65	Sep, 2015	30	87	31	Cluster C
ICDC-LM1782	M1-S65	Dec, 2015	30	87	31	Cluster C
ICDC-LM1784	M1-S65	Dec, 2015	30	87	31	Cluster C
ICDC-LM1233	M1-S42	Jan, 2015	30	87	32	nonclustered
ICDC-LM1203	M4-S51	Jan, 2015	331	87	33	nonclustered
ICDC-LM1497	M8-S14	May, 2015	30	87	34	nonclustered
ICDC-LM1502	M5-S33	May, 2015	30	87	35	nonclustered
ICDC-LM1218	M6-S15	Jan, 2015	30	87	36	nonclustered
ICDC-LM1620	M1-S67	Jul, 2015	345	87	37	nonclustered
ICDC-LM1625	M1-S65	Jul, 2015	30	87	38	nonclustered
ICDC-LM1659	M1-S65	Aug, 2015	27	87	39	nonclustered
ICDC-LM1520	M3-S99	May, 2015	317	87	40	nonclustered
ICDC-LM1175	M2-S5	Nov, 2014	30	87	41	nonclustered
ICDC-LM1197	M2-S15	Nov, 2014	30	87	42	nonclustered
ICDC-LM1204	M4-S77	Jan, 2015	315	87	43	nonclustered
ICDC-LM1373	M1-S51	Mar, 2015	331	87	44	nonclustered
ICDC-LM1361	M2-S80	Mar, 2015	30	87	45	nonclustered
ICDC-LM1296	M1-S58	Feb, 2015	323	87	46	nonclustered
ICDC-LM1220	M4-S102	Jan, 2015	324	87	47	nonclustered
ICDC-LM1492	M6-S28	May, 2015	30	87	48	nonclustered
ICDC-LM1572	M5-S13	Jun, 2015	322	87	49	nonclustered
ICDC-LM1665	M1-S42	Aug, 2015	30	87	50	nonclustered
ICDC-LM1848	M2-S83	Jan, 2015	30	87	51	nonclustered
ICDC-LM1674	M7-S16	Aug, 2015	27	87	52	nonclustered

### Whole-genome MLST analysis on selected L. monocytogenes isolates.

**FIG 1:**
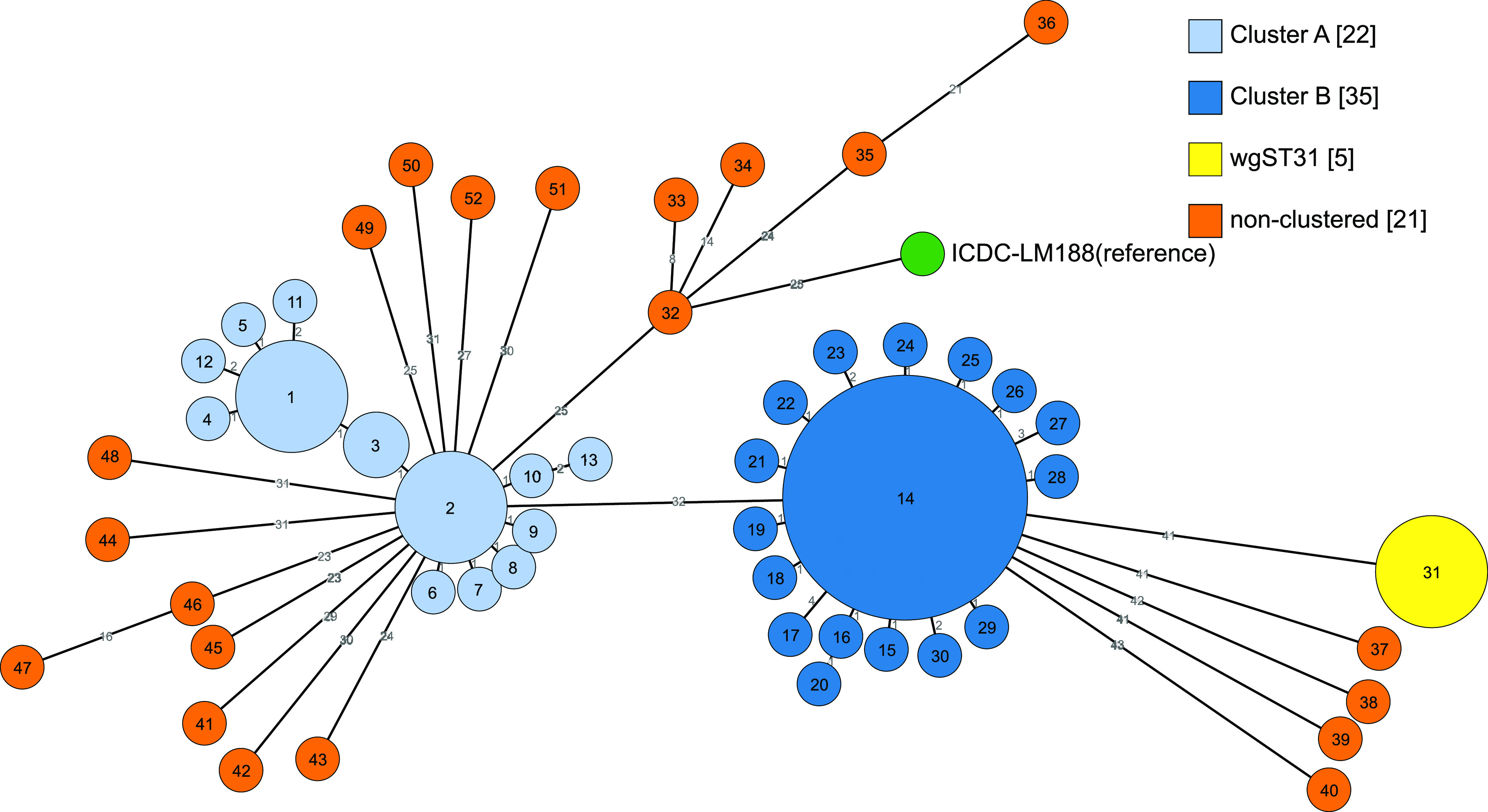
Minimum spanning trees based on CC87-wg-MLST allelic profiles present the relationship among the 83 CC87 L. monocytogenes isolates along with the reference strain ICDC-LM188. The number in each circle is wg-MLST sequence type (wgST), and the size of each circle is proportional to the number of isolates. The number of allelic differences between each wgST was labeled on the connecting line between two circles. Cluster A and Cluster B, which were two major groups of persistent isolates identified and are marked in light blue and dark blue, respectively. wgST31 is a unique single ST that contains more than one isolate and is marked in yellow. All the putative nonpersistent isolates are marked in orange. The reference strain ICDC-LM188 is marked in green.

Based on the classic seven-locus MLST scheme (6), our *in silico* analysis of the genomes divided the 83 isolates into two sequence types, with 48 ST87 isolates and 35 ST1166 isolates. The two STs belonged to CC87 and were separated by the *ldh* locus with one SNP difference. To provide further insight into the relationship and/or changes among these CC87 L. monocytogenes isolates, we performed whole-genome MLST (wgMLST) analysis of CC87 isolates, referred to as CC87-wg-MLST here to type all isolates. Note that we did not attempt to develop a CC87 wgMLST scheme and were only using this approach to obtain the best resolution for typing of the isolates rather than using the species-specific core genome MLST scheme (22). The gene-by-gene comparison was performed using the FAST-Gep program (23). A total of 2,723 shared loci and 378 polymorphic alleles were identified. The 83 isolates were typed into 53 wg-MLST types (wgSTs). The isolates were previously typed by PFGE, 86.7% of which belonged to the same PFGE type (PT30). CC87-wg-MLST showed a higher level of discrimination than PFGE. The 37 ST87-PT30 L. monocytogenes isolates were further divided into 24 wgSTs with three predominant types including wgST1 (*n* = 5), wgST2 (*n* = 5), and wgST31 (*n* = 5), while the 35 ST1166-PT30 isolates were further divided into 18 wgSTs with one predominant type, wgST14 (*n* = 19). All non-PT30 CC87 L. monocytogenes isolates were separated by CC87-wg-MLST with their specific types. Based on the CC87-wg-MLST allelic profiles, the minimum spanning tree was constructed with two clusters and 21 unclustered wgSTs identified (Fig. 1). Clusters were defined based on a cutoff of 4 alleles using the Silhouette index. The two clusters were named Cluster A (22 isolates) and Cluster B (35 isolates), with four alleles as the maximum pairwise allelic differences within each cluster. All except one unclustered wgST contained one isolate only. Unclustered wgST31 contained five isolates.

### Cluster A isolates caused persistent and cross contamination in raw meat stalls.

**FIG 2:**
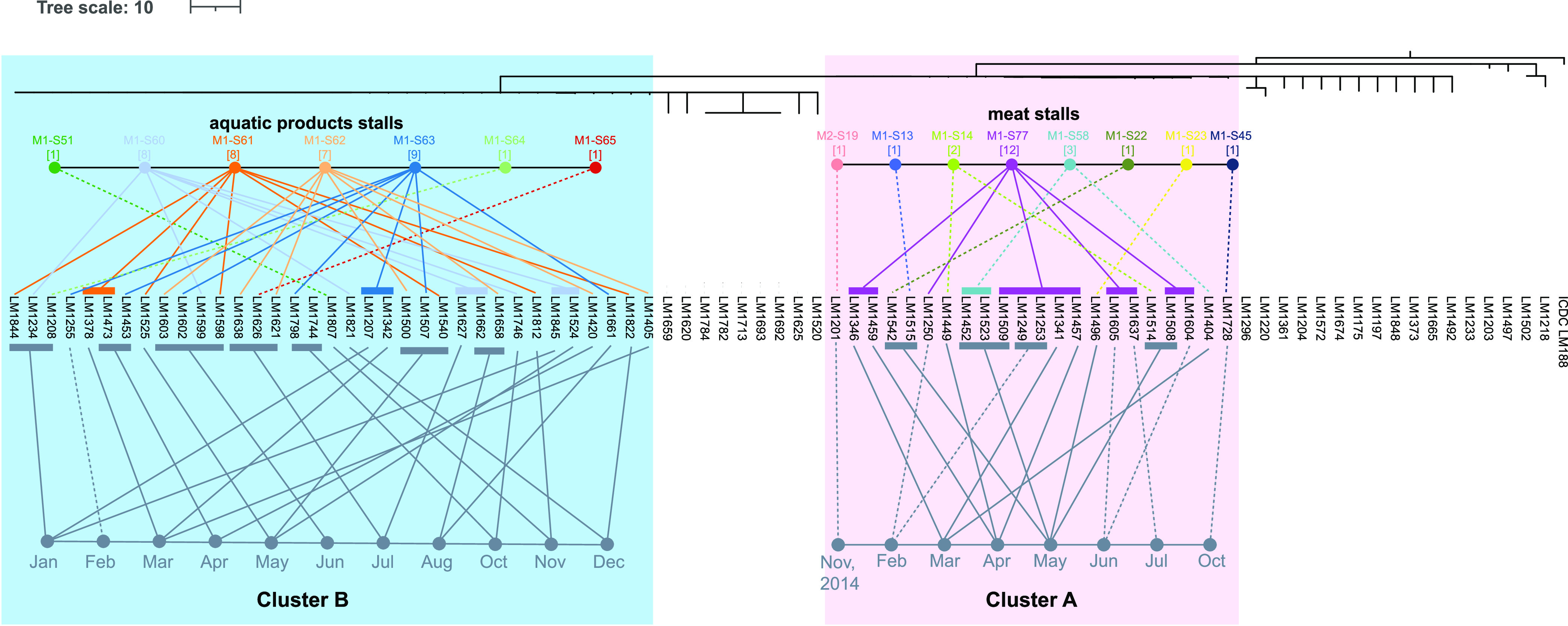
The dendrogram of hierarchical clustering of isolates based on CC87-wg-MLST allelic profiles. The metadata of Cluster A (in light pink shadow) and Cluster B (in light blue shadow) isolates, including isolating time and isolating stall, are mapped to the corresponding isolate.

All Cluster A L. monocytogenes isolates were isolated from the same retail market M1 during February to July and October 2015 (Table 1), with the exception of one isolate, ICDC-LM1201, being isolated from retail market M2 in November 2014. More than half (12/22) of Cluster A isolates were repeatedly isolated from the same stall (M1-S77) over a 6-month period (February to July, 2015) in a monthly sampling (Fig. 2). Moreover, 6 of these 12 isolates were detected in the meat grinder of the M1-S77 stall, which were separately isolated in a 6-month period. Additionally, five Cluster A isolates were separately isolated from five non-M1-S77 stalls in the M1 market in May 2015 (Fig. 2).

By CC87-wg-MLST, Cluster A isolates (all ST87-PT30) were divided into 12 types. wgST1 and wgST2 were the predominant types, which were linked with wgST3 by one allelic difference (Fig. 1). Isolate ICDC-LM1637, which had a different PFGE type (PT319), was identified as wgST11 and was isolated from stall M1-S77 in July 2015, which was the last sampling time point when L. monocytogenes was positive in this meat stall.

Among the Cluster A isolates, 13 out of 2,723 core genes were polymorphic based on CC87-wg-MLST (Table S2). In the 13 polymorphic genes, 10 were caused by SNPs, one caused by 1-bp in/del, and two caused by carrying different numbers of tandem repeats. An assembly-free whole genome SNP-calling strategy was further used to identify any extra SNPs outside the CC87-wgMLST genes and intergenic regions among the Cluster A isolates. A total of 12 SNPs were found with 10 being the same SNPs as identified by CC87-wg-MLST. Of these SNPs, eight were non-synonymous, two were synonymous, and two were in the intergenic regions. All the amino acid substitutions caused by nonsynonymous SNPs were predicted to be non-deleterious using software SIFT (24).

Pangenome analysis of Cluster A isolates using Roary predicted 2,952 hard-core genes (found in ≥99% of genomes), two soft-core genes (found in 95–99% of genomes), seven shell genes (found in 15–95% of genomes), and 70 cloud genes (found in <15% of genomes). Cluster A core-genome (2,952 genes) is 8.4% larger than the CC87 core-genome (2,723 genes). Interestingly, 66 of the 70 cloud genes were contiguous and belonged to a prophage, φtRNA-Arg, which was inserted into the downstream of tRNA-Arg in three Cluster A isolates, including ICDC-LM1201, ICDC-LM1523, and ICDC-LM1604. There were no SNP differences in the prophages φtRNA-Arg between ICDC-LM1201 and LM1523, both of which had two SNP differences from the isolate ICDC-LM1604. In addition, all Cluster A isolates harbored a 91 kb plasmid that was reported to be carried by a subset of ST87 isolates in our previous study (18).

### Cluster B isolates associated with persistence contamination in aquatic food products stalls.

The majority (91.4%) of Cluster B isolates were isolated from four neighboring aquatic products stalls (M1-S60 to M1-S63) in the M1 market, and the remaining four Cluster B isolates were isolated from three stalls, M1-S51(in November 2015), M1-S64 (in January 2015), and M1-S65 (in July and December 2015), which were located on the periphery of M1-S60 to M1-S63. All Cluster B isolates were ST1166-PT30.

By CC87-wg-MLST, 35 Cluster B isolates were divided into 17 wgMLST types, wgST14 contained 19 isolates while the other wgSTs had a single isolate. Among the 16 minor wgSTs, 11 wgSTs had one allelic difference from wgST14 (Fig. 1). Twenty polymorphic genes were identified and the gene functions tend to be related to metabolisms, such as the metabolism and transport of carbohydrates and inorganic ions (Table S3). Notably, two polymorphic genes encoded cell surface proteins were shared with the ones identified in Cluster A isolates.

By assembly-free wgSNP analysis, a total of 22 SNPs were identified among the 35 Cluster B isolates. Four SNPs were located in intergenic regions, while 18 SNPs were located in the coding genes, including 13 non-synonymous SNPs and five synonymous SNPs. One nonsynonymous SNP was located in the gene encoding a phage tail tape measure protein (ICDC-LM188 locus tag: A6K41_11830), which was not included in the alleles of the CC87-wg-MLST. All the amino acids substitutions caused by nonsynonymous SNPs were predicted to be non-deleterious using SIFT. In isolate ICDC-LM1420, one SNP resulted in a premature stop codon in the gene coding for PTS sorbose transporter subunit IIC (A6K41_00130 in ICDC-LM188). Pangenome analysis using Roary predicted 2906 hard core genes, 7 soft core genes, 7 shell genes and 5 cloud genes. All the Cluster B isolates harbored a prophage, which was inserted downstream the tRNA-Arg. The prophage showed no variation among the Cluster B isolates except for one isolate, ICDC-LM1525, which failed to be fully assembled as one contig. This prophage showed 92% identity and 84% coverage with the prophage φtRNA-Arg from a Cluster A isolate ICDC-LM1201. No plasmid was found in any of the Cluster B isolates.

### Characterizations of the five wgST31 isolates from aquatic food products stall.

Five isolates isolated from aquatic samples in the stall M1-S65 in September 2015 (*n* = 3) and December 2015 (*n* = 2) shared the same CC87-wg-MLST type (wgST31). Further assembly free wgSNP analysis found one SNP in an intergenic region and another synonymous SNP of a stop codon (locus tag in ICDC_LM188: A6K41_11850). The three isolates isolated in September 2015 showed different SNP profiles while the two isolates isolated in December 2015 shared the same SNP profile with one of the isolates in September. There was no prophage inserted into the end of tRNA-Arg in the wgST31 isolates. However, all five wgST31 isolates shared another prophage that was inserted between the FosX/FosE/FosI family fosfomycin resistance gene (locus tag in ICDC_LM188: A6K41_08870) and 23S rRNA (uracil-5-)-methyltransferase RumA gene (locus tag in ICDC_LM188: A6K41_08875), and was named φrumA here.

A novel plasmid, named pLM1692 was identified in all five wgST31 isolates. pLM1692 is about 60 kb in size and encodes 65 genes. Fifty-seven of the genes showed a high level of similarity with genes in a larger plasmid, pLM1686, as previously reported (Table S4) (19). In comparison with pLM1686, pLM1692 had a deletion of 30 kb which included a transposase gene, heavy metal resistance genes (*cadAC*), multicopper oxidase gene (*mco*), and copper transporter gene (*copB*).

### A new prophage φcomK inserted into *comK* and its association with persistent isolates.

Clusters A and B, as well as wgST31 isolates, were considered as persistent isolates while the remaining 21 isolates were considered as non-persistent isolates based on whether they were isolated multiple times or only once during the surveillance. The latter isolates had their unique wg-MLST profiles (from wgST32 to wgST52) with at least more than four allelic differences with each other (Fig. 1). These isolates all belonged to ST87 and 11 of the 21 isolates belonged to PT30.

Scoary was used to determine any associations between the accessory genome and the trait of persistence. With a naive *P* value of <0.05, there were no genes exclusively present in the genomes from all the persistent isolates. No genes were identified to be associated with persistence with 100% sensitivity and specificity. However, four genes located in a prophage (φcomK) were identified in the genomes of all the persistent isolates and some of the non-persistent isolates with 100% sensitivity and 41.67%~45.83% specificity, two of the genes were homologous to *lmo2321* and *lmo2322* (encoding gp44 [bacteriophage A118]) of the strain EGD-e with an amino acid sequence identity of 81% and 76% respectively, and the other two genes were annotated as hypothetical proteins.

We further characterized the φcomK in our isolates. Eighty-one of the 83 CC87 isolates of L. monocytogenes harbored the prophage φcomK, except for two putative nonpersistent isolates. The complete sequences of φcomK were available in 65 isolates, of which 58 isolates were persistent isolates and seven were putative non-persistent isolates. Seven types of φcomK were identified and were named as φcomK-type-1 to φcomK-type-7. The φcomK-type-1 prophages with the size of 40,671 bp were observed in all Cluster A isolates with 100% identity to each other. φcomK-type-2 prophages were observed in the isolates in Cluster B and wg-MLST profile type-3**1**. Interestingly, it was also found in a putative non-persistent isolate ICDC-LM1218, which was isolated from the market M6. φcomK-type-2 has a size of 38,304 bp. Two variants of φcomK-type-2 were found with 1-bp deletion and the other with one SNP. These two variants of φcomK were both carried by Cluster B isolates (ICDC-LM1207 and ICDC-LM1405). The φcomK-type-3 prophage with the size of 40,823 bp was harbored by two putative non-persistent isolates, ICDC-LM1203 and ICDC-LM1233, which were isolated from M4 and M1 markets respectively. The φcomK_type-5 to type 7 prophages were each harbored by one non-persistent isolate.

To investigate the diversity of these seven types of φcomK, the prophage sequences in each type were chosen for pan-genome analysis using Roary. Only one gene was identified as core gene (≥ 99% of the genomes), 137 genes were shell genes (15-95% of the genomes), and 64 cloud genes (<15% of the genomes). The Type 5 prophage was found to be the most diverse, sharing only one gene with other types of prophages (Fig. S2). In addition, 70 and 58 coding genes were predicted in φcomK_type-1 and φcomK_type-2 prophages, respectively, among which 24 genes that were annotated as hypothetical proteins were shared by both types of φcomK.

## DISCUSSION

The repeated introductions and the persistent survival of L. monocytogenes led to the contamination of food and food-associated environments, posing severe challenges to food safety. L. monocytogenes is a foodborne pathogen and should be closely monitored to prevent contamination in food production and distribution processes. Timely detection of contaminants and tracing the contamination routes are of great significance to the assurance of food safety. In the last decade, analyses of genome-scale data have been used to trace outbreaks-associated and persistent L. monocytogenes isolates with higher discriminatory power than conventional molecular subtyping methods, such as PFGE and MLST (11, 13). Whole-genome-based analysis can provide insight into more refined relationships and the dynamics of microevolution among a set of isolates in a certain niche. Further, it also can provide more genetic information on molecular determinants that are attributions of adaptation to stress and/or enhanced potentials (25). Several studies have focused on the characterization of persistent isolates over a long-term time frame with relative long-time intervals (13, 26). In this study, we studied a subset of CC87 L. monocytogenes isolates with the same PFGE patterns that were obtained from a 12-month surveillance project of retail food markets (21, 27), to further shed light on the persistent contamination, based on whole genome sequences, and identified two major persistent contaminations across the market. Whole-genome sequencing offered much higher resolution than PFGE as most isolates were the same PFGE type and were divided into two major clusters, allowing identification of contamination patterns and persistence of isolates.

### Persistent CC87 L. monocytogenes contamination events occurring in the studied market.

Whole-genome sequencing identified one persistent contamination event caused by Cluster A in the meat market. Genomic diversity was observed within Cluster A with 13 wgMLST types. All cluster A isolates were isolated from the same meat market M1 except for one isolate (ICDC-LM1201) from another market (M2) a few months earlier in November 2014. ICDC-LM1201 from market M2 was wgMLST Type-2, which was first detected in March 2015 in market M1, and three wgMLST types (Type-1, Type-3 and Type-6) were detected in February 2015 in market M1. Thus, Cluster A must have been circulating in different markets or upstream of a common source well before their first appearance in the markets. Interestingly, Cluster A was only detected once (based on PFGE type) in market M2 (21), but the reason of not persisting in M2 was unknown.

Stall M1-S77 was previously found to be repeatedly contaminated with the same PFGE type (21). Whole-genome sequence analysis provided a more detailed picture. For the 12 isolates sequenced, seven wgMLST types were found. These isolates were obtained from February 2015 to July 2015. It is possible that some of the genomic diversity was developed within the stall as wgST3, 4, 6, and 11 were found in that stall only. Stall M1-S77 had a meat grinder that was mainly used to grind meat from this stall and occasionally from other stalls. It was also likely that meat contaminated with Cluster A or other types entered the grinder and became a reservoir of continuous contamination as cleaning of the meat grinder may have been ineffective. There were unique wgSTs isolated in other stalls, that were not found in M1-S77, suggesting that the contamination of Cluster A in market M1 was widespread and was likely persisting in the entire meat market. Contamination of M1-S77 was likely to have been exacerbated by contamination of the meat grinder. It would be interesting to do further sampling to determine whether Cluster A remained in the market without intervention in the past few years.

The second persistent contamination event was caused by Cluster B isolates and had occurred in aquatic products and their related environments also in M1 market. Aquatic stalls selling live fish were in a different building level from the meat stalls in the same market. The contamination was concentrated around four stalls (M1-S60 to M1-S63) of aquatic products through the 12-month sampling period. Cluster B had only one predominant wgST (wgST14) along with 18 minority wgSTs. wgST14 was isolated over the 12-month sampling period, suggesting that it was well established in the stall environment. Since each stall had a fishpond to keep the fishes alive, it was highly likely Cluster B had established in the fishponds as a reservoir. Based on this observation, a real-time whole-genome sequencing monitoring for targeted cleaning and disinfection of the aquatic product stalls and fishponds, in particular in M1-S60 to M1-S63, could reduce or eliminate this persistent contamination.

There were five wgST31 isolates obtained in September and December 2015 from the M1-S65 stall, where one Cluster B isolate ICDC-LM1626 was isolated earlier in July. The latter differed from the wgST31 isolates by 41 alleles, suggesting two different contamination sources were independently introduced into M1-S65. wgST31 remained in the stall at least until the end of the year, while Cluster B was not recovered in this stall late in the year. We were unable to infer the causes of persistence of wgST31 in this stall.

### Mobile genetic elements (MGEs) and possible roles in the persistence and environmental adaptation of CC87 L. monocytogenes isolates.

ST87 L. monocytogenes has a highly stable genome backbone along with different prophages as major components of its accessory genome (18). MGEs may be involved in the adaptation and persistence of certain persistent L. monocytogenes clones. In this study, we found two new prophages (φcomK type-1 and φcomK type-2) in the persistent isolates. Cluster A isolates harbored Type-1 φcomK, and Cluster B isolates harbored two prophages, φtRNA-Arg and Type-2 φcomK, while wgMLST Type-31 isolates harbored two prophages, φrumA and Type-2 φcomK. Additionally, three Cluster A isolates, ICDC-LM1201, ICDC-LM1523, and ICDC-LM1604, harbored an additional prophage φtRNA-Arg, which was different from the prophage φtRNA-Arg in Cluster B isolates. These three isolates were of different wgSTs, suggesting that they acquired φtRNA-Arg prophages independently.

Moreover, scoary analysis identified four phage genes that were possibly associated with persistence. However, the functions of the genes were unknown and thus it is unknown whether they play any role in adaptation. The *comK* site was previously found to be a phage insertion site hot spot with different phages inserted at the site (28). The *comK* site has been found to play a regulatory role in niche adaptation and virulence (28–30). Insertion of a phage disrupts the *comK* gene, while excision of the prophage reverts *comK* to an intact and functional gene which encodes the master activator of the DNA uptake competence (Com) system (29). In this study, we found seven unrelated φcomK phages at the *comK* site. The insertion of multiple phages in the *comK* site may suggests that the Com system was quite frequently switched on and off through phage excision and insertion. The new phages may carry genes for adaptation to different environments.

Our previous study had reported that a 91-kb plasmid pLM1686 was carried by a subset of ST87 L. monocytogenes (18, 19). In this study, we found that the plasmid pLM1686 was exclusively carried by Cluster A isolates. The cadmium and some other heavy metal resistance genes might be necessary for the adaptation of Cluster A isolates in the meat products environments. Carriage of pLM1686 has been shown to enhance growth and biofilm formation, salinity tolerance, cell invasion, and cytotoxicity (20). Similar plasmids were also found in other STs and were found to contribute to tolerance against elevated temperature, salinity, acidic environments, oxidative stress, and disinfectants (31). The finding of pLM1686 carrying heavy metal resistance genes in persistent L. monocytogenes isolates suggests that the plasmid contributes to their persistence in the meat market environment. However, a smaller plasmid, 60-kb pLM1692, which was a truncated pLM1686 that has lost the cadmium, some other heavy metal resistance genes, and related transposable elements, was carried by the five wgST31 isolates isolated from the aquatic food or environments.

### Recommendations for control and prevention of L. monocytogenes contamination in retail markets.

The contamination of L. monocytogenes depends not only on the biological characteristics of bacterium itself, such as colonization ability, biofilm formation ability, and disinfectant resistance, but also on the living environments of the bacterium. The persistent contamination caused by Cluster A in this study was to a certain extent attributed to the hard-to-clean structure of the meat grinder. A combination of the repeated introduction of new strains and continuous survival in the grinder may have led to the persistent and cross contamination in meat stalls in the M1 market. Cluster B isolates were most likely surviving in the aquatic environments, which were live fish fishponds. Inadequate cleaning and disinfection of the fishponds were likely the main reason for the persistent survival of Cluster B isolates. The contrasting findings of two different L. monocytogenes clones surviving in two different environments underscore the importance of genomic analysis of the contaminating strains to understand the underlying mechanisms of contamination and targeted strategies for the prevention and control of L. monocytogenes transmission from the food processing chain to humans.

### Conclusions.

In summary, we identified three persistent subtypes of CC87 L. monocytogenes: (i) Cluster A isolates that persistently survived in a stall with a meat grinder, and possibly caused cross-contamination with other meat stalls in April and May 2015; (ii) cluster B isolates that persistently contaminated four aquatic food stalls throughout the year; and (iii) wg-MLST-Type-31 isolates that exclusively contaminated one aquatic food stall. Although no clear genetic determinants of persistence were identified, the mobile genetic elements, including prophages and plasmids, were identified to be cluster-specific, likely contributing to different fitness and adaptation in the complex ecosystems. Our study showed that the application of whole-genome sequence analysis can significantly inform food safety surveillance of L. monocytogenes and control efforts in retail markets.

## MATERIALS AND METHODS

### DNA Extraction, whole-genome sequencing, assembly, and annotation.

DNA extraction was performed using the Wizard Genomic DNA purification kit (Promega corporation, 2800 Woods Hollow Road, Madison, WI 53711 USA), according to the manufacturer’s instructions for Gram-positive bacteria. Total DNA obtained was subjected to quality control by agarose gel electrophoresis and quantified by Qubit. A pair-end library with an insert size of 500 bp was constructed and sequenced using an Illumina HiSeq X by PE150 strategy at the Beijing Novogene Bioinformatics Technology Co, Ltd. After removing the low-quality reads and adapter reads, the filtered reads were assembled by SOAPdenovo v.1.05 to generate scaffolds, and then annotation was performed using the NCBI Prokaryotic Genomes Automatic Annotation Pipeline (PGAAP).

### Whole genome analysis.

The seven-locus MLST was performed *in silico* using the BIGSdb-Lm database (https://bigsdb.pasteur.fr/listeria/). A genome-by-genome allele calling program without a containing scheme, named CC87-wg-MLST here, was performed using the Fast-Genome Profile (Fast-GeP), which is freely available from https://github.com/jizhang-nz/fast-GeP (23). The minimum spanning trees were constructed using the software GrapeTree v.1.5.0 with the MSTreeV2 method to present the relationship among the isolates based on whole genomic scale allelic profiles (32). The different sequences of all the shared loci among the isolates were assigned different alleles, and the combination of the alleles of the shared loci defined the whole genome sequence type (wgST). The core/pan-genome analyses were performed using Roary v.3.13.0 (33) with default parameters (the minimum percentage identity for blastp set to 95, and percentage of isolates a gene must be in to be core set to 99). The general feature format 3 (gff3) files generated by Prokka v1.14.4 (34) were used as input data for Roary. According to the harboring rate of each gene, all the pan genes were classified as hard-core genes (in ≥99% of genomes), soft-core genes (in 95–99% of genomes), shell genes (15–95% of genomes), and cloud genes (in <15% of genomes). Core genome SNPs calling was performed using Snippy v.4.4.5 (https://github.com/tseemann/snippy).

### Analysis of prophages and plasmids.

Prophages were identified using the online webserver PHASTER (http://phaster.ca) and were compared by BLASTN among each type of prophages. To investigate the diversity of prophage φcomK in CC87 isolates, Roary was used with the options of -i (minimum percentage identity for blastp) and -cd (percentage of isolates a gene must be in to be core) of 96 and 99 respectively. The plasmids in the isolates were identified by BLASTN searches using the sequence of pLM1686 as a query against each assembled sequence. The filtered reads were mapped to the sequence of pLM1686 plasmid using BWA v0.7.17, when scaffolds in some isolates only aligned to partial of pLM1686.

### Data availability.

The 60 L. monocytogenes genome assemblies sequenced in this study were deposited at DDBJ/ENA/GenBank under the BioProject accession no. PRJNA795564. The other 23 genome assemblies sequenced in our previous study were deposited at DDBJ/ENA/GenBank under the BioProject accession no PRJNA447903. The genome accession numbers for each isolate used in this study were listed in Table S4 in the supplemental material.

## Supplementary Material

Reviewer comments
