# Supplementary material for: Dissecting Listeria monocytogenes Persistent Contamination in a Retail Market Using Whole-Genome Sequencing
Source: Microbiol Spectr. 2022 May 17;10(3):e00185-22. doi: 10.1128/spectrum.00185-22 (PMC9241689; doi:10.1128/spectrum.00185-22)
Supplement: SUPPLEMENTAL FILE 5 — Supplemental material. Download spectrum.00185-22-s005.pdf, PDF file, 0.4 MB [file spectrum.00185-22-s005.pdf]

Stall ID	Sample kinds	Months											
		Jan	Feb	Mar	Apr	May	Jun	Jul	Aug	Sep	Oct	Nov	Dec
M1-S51	viscera		86	331	54	54				54		30*	
M1-S60	viscera	30*							30*		30*		
M1-S60	pond water				9			30*			30*		30*
M1-S60	subway water	30*				30*	30*						
M1-S61	viscera					30*						11	
M1-S61	pond water	30*		30*	30*	30*	30*						30*
M1-S62	pond water			30*	30*	30*	30*	30*	30*		30*		
M1-S63	pond water	30*	30*	30*	30*	30*	30*	30*	30*		30*		
M1-S63	viscera		30*						30*		30*	30*	
M1-S64	viscera							340					
M1-S64	pond water	30*											
M1-S65	viscera							30*		30			30
M1-S65	pond water			30*			38			30			30
M1-S65	environmental swab							30	27	30			30
M1-S66	pond water			86	347			47					
M1-S67	viscera			11									
M1-S67	pond water							345					

Fig S1. Presence of *L. monocytogenes* isolates in aquatic stalls during the 12-month survey. Sampling sites contaminated by *L. monocytogenes* more than once were included. Different PFGE types (PTs) were represented by different colors as shown. PT30 included two STs. PT30 and PT30* referred to ST87 and ST1166, respectively

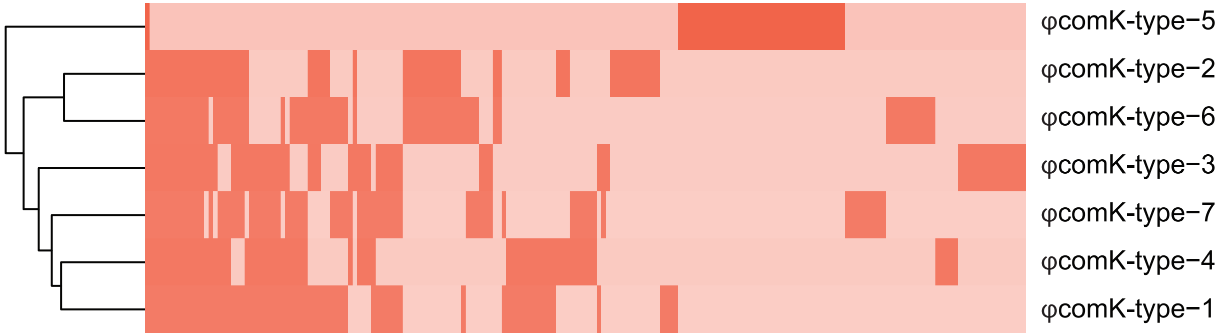

Fig S2. The presence and absence of core and accessory genes from seven types of prophage φcomK in this study.
